# Setting the pace: the 2011 Australasian Podiatry Council conference

**DOI:** 10.1186/1757-1146-4-20

**Published:** 2011-07-15

**Authors:** Hylton B Menz, Julia Firth

**Affiliations:** 1 Musculoskeletal Research Centre, Faculty of Health Sciences, La Trobe University, Bundoora, Victoria, Australia; 2 Podiatry Department, St Vincent's Hospital, Melbourne, Victoria, Australia

## Abstract

The 2011 Australasian Podiatry Council conference was held from April 26 to 29 in Melbourne, Victoria, Australia. This commentary provides a brief overview of the conference, including the speakers and topic areas covered, selected original research highlights, and award winning presentations.

## Background

The 2011 Australasian Podiatry Council conference (APC2011) was held at the Melbourne Exhibition and Convention Centre from April 26 to 29, and attracted over 1,000 delegates. The purpose of this commentary paper is to provide an overview of the conference, including the speakers and topic areas covered, selected original research highlights and award winning presentations.

### Speakers and topics covered

APC2011 featured three international keynote speakers - Professor Andrew Boulton (University of Manchester, UK), Professor Irene Davis (Harvard University, USA) and Dr Edward Roddy (Keele University, UK) - and 10 invited speakers (see Table 1). The speakers attracted considerable media attention, and interviews with Prof Irene Davis and Trevor Prior conducted during the conference can now be viewed at the *JFAR *YouTube channel [1].

**Table 1:** Invited speakers

Speaker	Institution
Dr Harvinder Bedi	Orthosports Victoria, Melbourne
Prof Peter Brooks	Australian Health Workforce Institute, Melbourne
Dr Kay Crossley	University of Melbourne
Dr George Koulouris	Melbourne Radiology Clinic
Dr Stephen Marty	Monash University/Pharmacy Board of Australia
A/Prof Bill McGuiness	La Trobe University, Melbourne
Trevor Prior	Premier Podiatry, London, UK
Dr Monique Ryan	Royal Children's Hospital, Melbourne
A/Prof Jonathan Shaw	Baker IDI Heart and Diabetes Institute, Melbourne
Jason Warnock	Podiatry Board of Australia
A/Prof Scott Wearing	Bond University, Queensland

**Figure 1:**
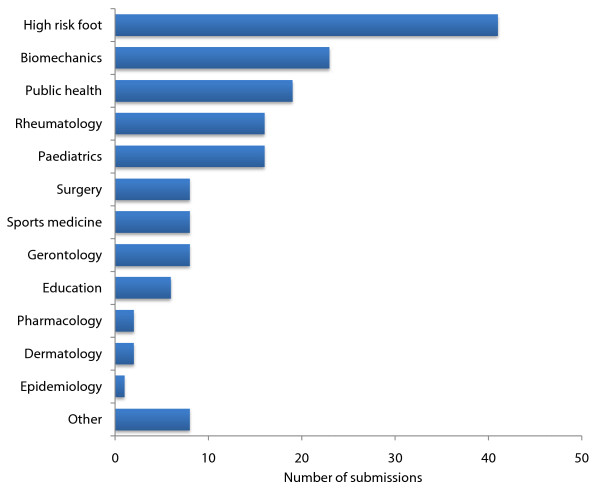
**Breakdown of abstracts submitted by topic area**.

In addition to the keynote and invited speaker presentations, a total of 158 free papers were submitted for consideration - the largest number of submissions ever received for an Australasian podiatry conference. A breakdown of abstracts by topic area is shown in Figure 1, which highlights the dominance of research activity in the fields of the high risk foot management and foot and ankle biomechanics. Of these abstracts, 54 were accepted for oral presentation and 61 for poster presentation. Each of these abstracts can be freely downloaded as html text or as a PDF file at the *JFAR *website [2]. Of particular note, the conference program featured the largest number of presentations of systematic reviews [3-8] and randomised trials [9-13] indicating the increasingly high quality of research being conducted in Australasia.

**Figure 2:**
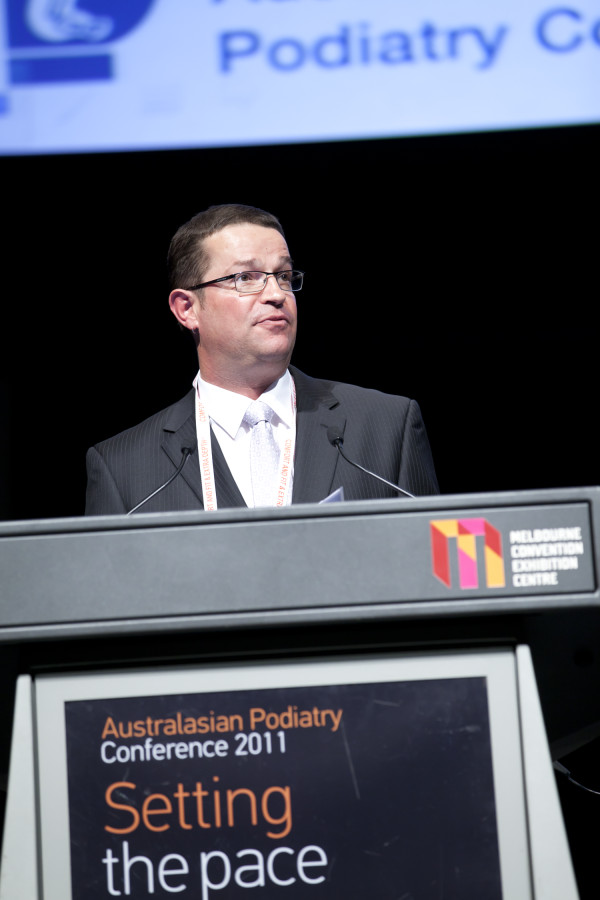
**Australasian Podiatry Council president Andrew Schox opens the conference**.

**Figure 3:**
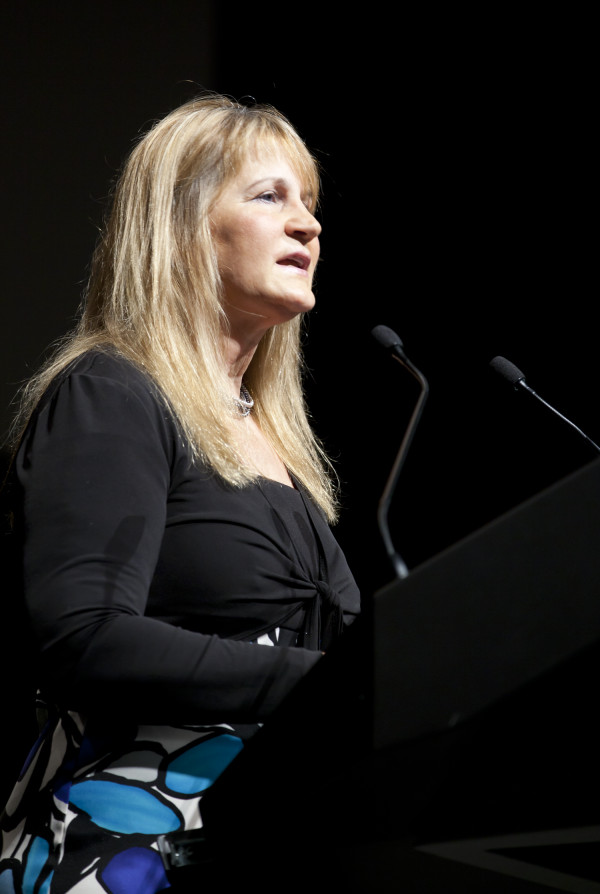
**Keynote speaker Professor Irene Davis**.

**Figure 4:**
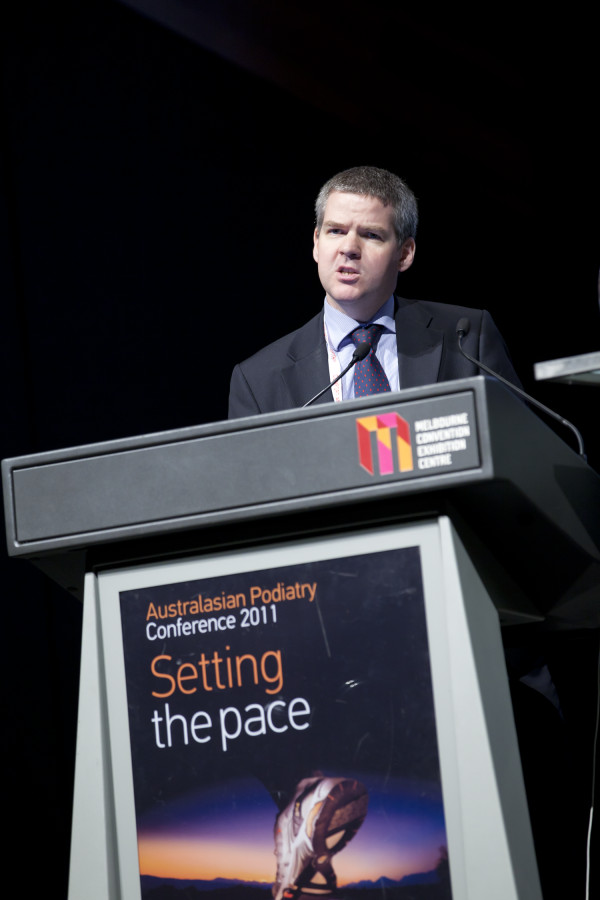
**Keynote speaker Dr Edward Roddy**.

**Figure 5:**
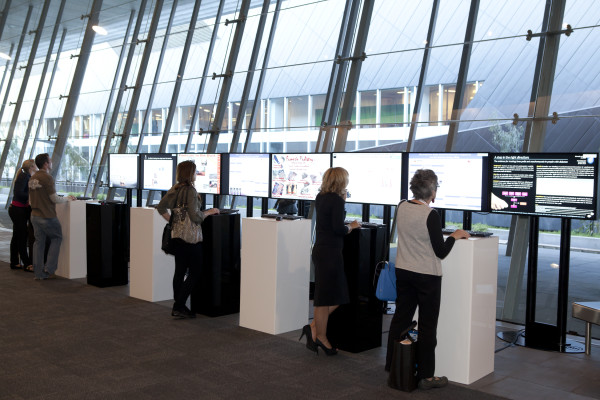
**The virtual poster display area**.

APC2011 also saw the introduction of 'virtual' poster displays using flat-screen monitors, which enabled the presentation of substantially more posters than the traditional hard copy approach. Photos from the conference are provided in Figures 2, 3, 4 and 5.

### Selected original research highlights

APC2011 featured more original research papers than any previous Australasian podiatry conference. Selected research findings for each of the conference theme areas are provided below.

#### High risk foot

• Hospital separations for diabetes-related foot disease in Victoria, Australia, vary according to the degree of socioeconomic disadvantage of each local government area [14]

• Standardising diabetic foot management by instituting multi-disciplinary teams, clinical pathways, telehealth support and surveys resulted in a 64% reduction in amputation rates and a 24% reduction in average length of stay in Queensland hospitals [15]

#### Rheumatology

• Ultrasound-guided injection of dexamethasone is more effective than placebo (sterile saline) in the treatment of plantar fasciitis [11]

• Intra-articular injection of hyaluronan (Synvisc^®^, hylan G-F 20) is no more effective than placebo (sterile saline) in the treatment of pain associated with first metatarsophalangeal joint osteoarthritis [13]

#### Gerontology

• A multifaceted podiatry intervention consisting of foot orthoses, footwear advice and a foot and ankle exercise program can reduce falls by 36% in older people [12]

#### Paediatrics

• Traditional school shoes restrict children's foot motion during walking, particularly at the midfoot, during the contact period and propulsion phases of gait [16]

• A 'Toe-Walking Tool' has been developed and validated as a comprehensive way to assess children with idiopathic toe-walking and to assist in appropriate referral [17]

#### Biomechanics and foot orthoses

• Increasing plantar sensory feedback to the medial aspect of the foot reduces midfoot pronation during walking [18]

• Lateral wedged insoles are no more effective than flat inserts in the treatment of medial compartment knee osteoarthritis [9]

#### Wound management

• A one-year audit of microbiology culture results in a high risk foot clinic indicated a surprisingly high incidence of Methicillin-resistant Staphylococcus aureus (16% of patients) [19]

• Negative pressure wound therapy achieves complete wound closure in 60% of patients with post-surgical wounds and is associated with a lower rate of major amputation [20]

#### Surgery

• A meta-analysis of 24 studies indicates that the scarf osteotomy produces a marginally greater reduction in the 1-2 intermetatarsal angle in patients with hallux valgus compared with the chevron osteotomy [5]

### Conference award winners

The APC2011 Conference Awards Committee consisted of Dr Karl Landorf (Chair), Dr Angela Evans, Mario Horta and Prof Keith Rome. Four awards were presented at the conference: (i) the *JFAR Best Paper Award*, (ii) the *Best New Investigator Award*, (iii) the *Best Non-Research Paper Award *and (iv) the *Best Research Poster Award*. A summary of the award-winning presentations is provided below, and abstracts of each of the award-winning presentations can be downloaded at the *JFAR *website by following the relevant links in the reference list.

### *JFAR *Best Paper Award

This was awarded to Dr Shan Bergin, Southern Health, for her presentation "Diabetes related foot disease: know thine enemy" [14]. This paper highlighted the fact that significant numbers of people in Victoria with diabetes suffer from neuropathy, PVD, ulcers and amputation, and that the socio-economic status of patients' local area can have a significant impact on clinical outcomes. A full paper based on this presentation has recently been published in *JFAR *[21].

#### Best New Investigator Award

This was awarded to Dr George Murley, La Trobe University, for his presentation "Do foot orthoses change lower limb muscle activity in people with flat-arched feet towards a pattern observed in those with normal-arched feet?" [22]. Derived from his recently published PhD studies, this presentation showed that the electromyographic (EMG) activity of lower limb muscles differs in people with flat feet, and that foot orthoses can alter these muscle activation patterns.

#### Best Non-Research Paper Award

This was awarded to Sylvia McAra, Charles Sturt University, for her presentation "Glyceryl trinitrate therapy for ischaemia, painful diabetic neuropathy, healing of foot ulceration and other podiatric conditions: a literature review" [23]. Glyceryl trinitrate is widely known to be a very effective vasodilator for the treatment of angina, but may also facilitate wound healing with a mechanism of action similar to hyperbaric oxygen therapy. This presentation provided a comprehensive overview of the topical application of this therapy for wound management.

#### Best Research Poster Award

This was awarded to Gordon Hendry and colleagues, Glasgow Caledonian University, for the poster "Foot related impairments and disability in juvenile idiopathic arthritis persist despite modern day treatment paradigms" [24]. This poster was based on a cross-sectional survey of foot related impairments and disability in patients with juvenile idiopathic arthritis, and showed that approximately two thirds of the sample had mild to moderate foot-related disability, despite being managed with disease modifying anti-rheumatics, anti-TNF drugs and specialist paediatric podiatry care.

## Summary

APC2011 was a highly successful event that showcased the breadth and quality of clinical foot and ankle research being conducted in Australasia. The next Australasian podiatry conference will be hosted in Sydney in 2013.

## Competing interests

The authors declare that they have no competing interests.

## Authors' contributions

HBM drafted the manuscript and JF provided feedback. Both authors read and approved the final manuscript.
